# Latent Structure and Item Functioning of Self-Referent Encoding Task Word Stimuli in Preadolescent Youth

**DOI:** 10.1177/10731911241289249

**Published:** 2024-11-23

**Authors:** Lindsay N. Gabel, Thomas M. Olino, Brandon L. Goldstein, Daniel N. Klein, Kasey Stanton, Elizabeth P. Hayden

**Affiliations:** 1 Western University, London, ON, Canada; 2 Temple University, Philadelphia, PA, USA; 3 UConn Health Center, Farmington, CT, USA; 4 Stony Brook University, NY, USA; 5 University of Wyoming, Laramie, WY, USA

**Keywords:** Self-Referent Encoding Task, self-concept, factor analysis, item response theory, childhood

## Abstract

The Self-Referent Encoding Task (SRET) can be used to measure self-concept via endorsement of trait words, a robust metric associated with depression severity. Our study is the first to investigate the structural validity and item functioning of SRET endorsement scores using confirmatory factor analysis and item response theory. Community-dwelling preadolescent youth (*N* = 508; *M*_age_ = 12.39 years, *SD*_age_ = .72) were shown a list of positive and negative trait adjectives and made binary ratings of whether words were self-descriptive. The SRET exhibited a two-factor structure, comprising positive and negative factors. Positive items were endorsed by most children and best estimated information about positive self-concepts below average levels of positivity. Conversely, negative items were unendorsed by most children and best estimated information about negative self-concepts above average levels of negativity. We identify standardized, psychometrically sound, and developmentally sensitive SRET items for assessing youth self-concept and its associations with depression risk.

Cognitive diatheses have long been viewed as central to the development and maintenance of depression (Beck, 1967; Ingram et al., 1998). Prevailing cognitive models of depression implicate maladaptive self-referential processing as a key vulnerability in the etiology of depressive symptoms (Beck, 1967; Phillips et al., 2010). Indeed, decades of work have shown that self-referential processing is negatively biased in depressed populations, as well as healthy individuals at risk for depression (LeMoult & Gotlib, 2019). In addition, evidence suggests that depressive self-referential processing is characterized by differential processing of positive and negative stimuli (Johnson et al., 2007), such that depressed and at-risk individuals incorporate *more* negative and *less* positive information in constructing beliefs about themselves (Beck, 2008).

To date, the literature on self-schemas and depression has primarily focused on adults and adolescents, although evidence suggests that maladaptive self-schemas emerge in early childhood (Abela & Hankin, 2008; Hayden et al., 2013; Ingram et al., 1998) and precede the development of depression (Hayden et al., 2013; LeMoult & Gotlib, 2019). Examining links between children’s self-schemas and depressive symptoms in childhood may therefore elucidate processes by which maladaptive self-schemas potentiate risk for later depression. More specifically, studying these phenomena in late childhood (i.e., ages 9–12 years) may be particularly illuminating. The temporal positioning of this developmental stage between middle childhood, a time when children’s sense of self begins to stabilize (Abela & Hankin, 2008; Orth et al., 2021), and adolescence, a period defined by substantial within-person changes in self-concept (Molloy et al., 2011; Orth et al., 2021) and increased depression risk (Thapar et al., 2012), renders it well suited to studying developmental mechanisms of cognitive vulnerability to depression.

Examining links between early self-schemas and later depressive symptoms requires valid and developmentally sensitive measurement tools for assessing cognitive vulnerability in children. The Self-Referent Encoding Task (SRET; Derry & Kuiper, 1981), a behavioral task assessing biases in processing self-referent information, is one of the most widely used experimental paradigms for evaluating cognitive vulnerability to depression. In this task, individuals make binary decisions (i.e., yes/no) about whether multiple positive and negative trait adjectives (e.g., *smart*, *foolish*, *brave*) describe themselves (Derry & Kuiper, 1981). Several metrics can then be calculated, including the number of words endorsed, number of words recalled in a subsequent incidental recall task, processing scores (i.e., the proportion of words endorsed *and* recalled to words endorsed), and reaction time for deciding whether words are self-descriptive (Dainer-Best et al., 2018; Hayden et al., 2014; P. Liu et al., 2023). The SRET was initially developed for use with adults and to date has been used primarily with adults (e.g., Derry & Kuiper, 1981; Dobson & Shaw, 1987) and adolescents (e.g., Auerbach et al., 2015; Black & Pössel, 2013; Connolly et al., 2016; Pössel et al., 2006; Timbremont & Braet, 2004). However, developmentally sensitive versions of the SRET have been developed for use with youth as young as 6 years (Gençöz et al., 2001; Goldstein et al., 2015; Hammen & Zupan, 1984; Hayden et al., 2013, 2014; P. Liu et al., 2023; Taylor & Ingram, 1999; Timbremont & Braet, 2004). These child-friendly versions were developed using rational methods to accommodate a Grade 3 reading level, with positive and negative words matched for frequency of use in written literature (Goldstein et al., 2015; Hammen & Zupan, 1984; Hayden et al., 2006).

Of the SRET metrics, processing scores (i.e., the proportion of all endorsed positive or negative words recalled) are the most widely used in the extant literature (P. Liu et al., 2023). However, because processing scores amalgamate multiple items, they cannot be examined via factor analysis and analyses of item functioning. In contrast, word endorsement scores are amenable to such analyses and show particularly strong associations with depression severity (Dainer-Best et al., 2018). For these reasons, we focus on word endorsement, which is conceptualized as a measure of self-concept, or individuals’ overarching view of themselves and their qualities (Butler & Gasson, 2005; Crone et al., 2022). As with self-schemas, self-concepts emerge early in development (Eder & Mangelsdorf, 1997; Harter, 2012) and are associated with risk for psychopathology across the lifespan (Hayden et al., 2013; Orth et al., 2008, 2009).

As an index of children’s self-concept, the SRET has several advantages. Notably, it balances comprehensiveness with brevity. The most common extant measures of youth self-concept require children to read, interpret, and respond to anywhere from 10 to 150 statements (Butler & Gasson, 2005). In contrast, the single-word items of the SRET require a fraction of the time and cognitive sophistication needed to digest sentence-format items, enabling children to complete more items in less time. Indeed, the simplicity of the SRET renders it well suited to the assessment of self-concept in child populations, for whom adult measures are less appropriate given developmental differences between the developing and mature self-concept (Crone et al., 2022; Guerin & Tatlow-Golden, 2019; Marsh et al., 2005). The efficiency of its items also permits the SRET to maximize content validity by including items that are relevant for the multiple domains that make up youth self-concept (e.g., peers/popularity, school/academic, parent/family, physical appearance, etc.), which are inconsistently mapped in other measures (Guerin & Tatlow-Golden, 2019).

Despite widespread use of the SRET, however, its measurement properties have gone largely unexamined. In particular, no study has yet examined the structural validity or item functioning of the SRET in any population, youth or adult. Recent years have seen calls for a renewed focus on the measurement of psychological phenomena as a means of addressing critical issues that plague the field, such as the replication crisis (Hayden, 2022; Parsons et al., 2019). Indeed, as summarized by others, psychological science “rests on the foundations of measurements” (Parsons et al., 2019). In the absence of this critical measurement work, the validity and utility of the SRET for assessing cognitive vulnerability to depression across development remain unclear.

Metrics yielded by the SRET and widely used in research, such as the number of positive and negative words endorsed, are aggregated on the assumption of a two-factor structure, with individuals’ positive and negative self-concepts representing distinct dimensions (Derry & Kuiper, 1981; Hammen & Zupan, 1984; Hayden et al., 2006). However, it is also possible that positive and negative self-concept occupy opposing ends of a single spectrum, such that SRET items load onto a single factor. To date, however, whether the SRET conforms to a one- or two-factor structure remains unexplored. Because the SRET has traditionally been used to disentangle the contributions of positive versus negative self-schemas (or self-concepts) to depression vulnerability, establishing the latent structure of the SRET is critical for determining its validity and utility in research on youth cognitive vulnerability to depression.

Additional limitations of the extant SRET literature include the lack of standardized stimuli and limited understanding of item functioning. These limitations have produced a literature of idiosyncratic SRETs whose positive and negative items vary substantially in content and number, rendering direct comparisons of SRET indices across studies challenging. In addition, the extent to which SRET items (a) map children’s latent positive and negative self-concept and (b) effectively discriminate between individuals across the full range of positive/negative self-concept is unknown. Exploring the utility of SRET items is necessary for informing refinement strategies, such as discarding poorly functioning items and identifying unmapped areas of the latent construct for which new items may be needed. In summary, there is a need for standardized, psychometrically sound, and developmentally sensitive SRET items for assessing preadolescent children’s self-concepts.

## The Present Study

To this end, we examined the structural validity and item utility of a developmentally sensitive SRET in a large community sample of preadolescent children. Our aims were twofold. First, we aimed to establish the factor structure of a developmentally sensitive version of the SRET using confirmatory factor analysis (CFA). Based on theory used to develop SRET items (Derry & Kuiper, 1981; Hammen & Zupan, 1984), we hypothesized that the SRET would exhibit a two-factor structure. Second, we aimed to explore the utility of SRET word stimuli using item response theory (IRT). Specifically, we examined the degree of item fit, the probability of endorsing a given word based on a child’s underlying positive/negative self-concept (i.e., item response patterns), and the amount of information provided by items and the overall scale about a child’s underlying positive/negative self-concept (i.e., item and test information). Together, our analyses speak to the validity and utility of SRET items as measures of children’s positive and negative self-concepts.

## Method

### Participants

Participants were drawn from two separate samples recruited from different geographic locations: the Stony Brook area of the Northeastern United States (U.S. sample; *N* = 430) and Southwestern Ontario in Canada (Canadian sample; *N* = 78). Both samples were recruited from larger longitudinal studies occurring in each location.

#### U.S. Sample

A total of 430 children (*M*_age_ = 12.63 years, *SD*_age_ = .43; 225 boys) were recruited from the Stony Brook area of New York. The sample was predominantly White (89.3%), with the remainder of the sample identifying as Black (7.7%), Asian (2.6%), Native American (0.2%), or Other (0.2%). In addition, 20.5% of the sample identified as Hispanic or Latino. The sample was largely working- to middle-class for the geographic area, with 59.1% of the sample reporting an annual household income of $70,000 USD or greater, 21.6% reporting $40,000 to less than $70,000, 4.0% reporting $20,000 to less than $40,000, and 1.6% reporting less than $20,000 at study entry (age 3). In total, 13.7% of the sample was either unsure or declined to provide family income data. Children possessed average verbal ability based on Peabody Picture Vocabulary Test (PPVT; Dunn & Dunn, 1997) scores obtained at age 3 (*M* = 103.37, *SD* = 13.34).

#### Canadian Sample

A total of 78 children (*M*_age_ = 11.12 years, *SD*_age_ = .64; 44 boys) were recruited from Southwestern Ontario in Canada. The sample was predominantly White (97.4%), with the remainder of the sample identifying as Black (1.3%) or Other (1.3%). No participants identified as Hispanic or Latino. The sample was largely middle-class or higher, with 50.0% of the sample reporting an annual household income of $70,000 CAD or greater, 25.6% reporting $40,000 to less than $70,000, 12.8% reporting $20,000 to less than $40,000, and 3.8% reporting less than $20,000 at study entry (age 3 years). In total, 7.7% of the sample was either unsure or declined to provide family income data. Children possessed average verbal ability based on PPVT scores obtained at age 3 years (*M* = 102.9, *SD* = 13.9).

We compared the two samples on demographic variables and SRET item endorsement using chi-square and independent samples *t*-tests. The samples did not differ significantly by sex or PPVT score. The U.S. sample was significantly older than the Canadian sample, *t*(90.11) = 20.06, *p* < .001; however, children in both samples were within a range of 4.5 years of age and thus within the same developmental stage. The U.S. sample also had significantly more participants identifying as non-White and/or Hispanic/Latino, χ^2^(1) = 14.51, *p* < .001, reflecting differences in the demographic makeup of the geographic areas from which the samples were recruited. Direct comparisons of family income across samples were not possible due to differences in currency value, although the proportions of participants falling within each income band were similar across samples. Given minimal differences between the samples and SRET procedures, we combined the two samples to ensure a sample size of at least 500 participants (total *N* = 508, overall *M*_age_ = 12.39 years, *SD*_age_ = .72, 269 boys),^1^ the recommended benchmark for CFA and IRT analyses with data sets similar to ours (Morizot et al., 2007; Muthén & Muthén, 2002).

### Measures and Procedure

#### Mood Induction Procedure

Children and their primary caregivers were invited to a laboratory visit, which began with caregivers completing consent for their children and children completing assent with a postbaccalaureate experimenter. The same SRET administration procedures were used across the two samples. Prior to administration of the SRET, children completed a sad mood induction procedure to activate latent cognitive vulnerability (Taylor & Ingram, 1999).^2^ Film clips are among the most effective stimuli for inducing emotion in laboratory settings with youth (Joseph et al., 2020), and similar mood induction procedures have been used in previous studies using the SRET (e.g., Hayden et al., 2006). Children viewed one of several sad film clips (i.e., a scene from *My Sister’s Keeper*, *Marley & Me, 50-50*, *Champ*, or *Dead Poets Society*) and subsequently listened to 10 minutes of a looped musical piece (*Adagio for Strings*; Barber, 1936) shown to evoke sadness (Siemer, 2005). To assess changes in mood before and after the mood induction, we administered a manipulation check in which children completed pre- and post-ratings of their mood using a 5-point ordinal scale (1 = *very sad*; 5 = *very happy*). Numerical ratings were accompanied by verbal and visual descriptors (i.e., words and pictures of facial expressions) to facilitate children’s understanding of the scale. A comparison of children’s pre- and post-ratings indicated that the mood induction was effective in eliciting sad mood, *t*(506) = 46.86, *p* < .001; *M*_pre_ = 3.89 (.70), *M*_post_ = 1.75 (.76).

#### SRET

Children’s positive and negative self-concepts were assessed using the SRET (Derry & Kuiper, 1981), a widely used behavioral task assessing biases in processing self-referent information. Children were presented with 12 positive adjectives (e.g., *smart*, *brave*, *friendly*) and 11 negative adjectives (e.g., *selfish*, *foolish*, *ugly*) and asked to make binary decisions (i.e., yes/no) about whether each word described themselves. We used developmentally sensitive word stimuli developed by others (Hammen & Zupan, 1984; Hayden et al., 2006). Words were selected for Grade 3 readability, with the mean age of acquisition being 5.62 years (mean *SD* = 2.09) and ranging from 3.24 to 8.25 years (Kuperman et al., 2012). Positive and negative words were matched for word frequency in the English language (Hayden et al., 2006). Word stimuli were presented one at a time in random order for each child using handheld flash cards, with words read aloud by the examiner (U.S. sample), or E-Prime computer software, with words read aloud by a computer (Canadian sample). Four neutral words (i.e., *big*, *old*, *young*, *short*) were presented alongside the positive and negative item set—two at the beginning and two at the end of the task—to reduce primacy and recency effects. After a brief delay, children were then presented with an unexpected incidental recall task for the purposes of calculating additional SRET metrics not used in the present study (i.e., processing scores). Given recent work showing that word endorsement is among the most robust of the SRET metrics in predicting depressive symptoms (Dainer-Best et al., 2018), as well as our interest in examining factor structure and item functioning, we examined the total number of positive and negative words endorsed, respectively. Thus, higher positive and negative item endorsement scores (hereafter referred to as SRET scores) indicated higher positive and negative self-concepts, respectively. At the end of the study session, children were given gift cards for their participation. Study procedures were approved by the Western University Research Ethics Board and the Stony Brook University Institutional Review Board.

#### Depressive Symptoms

Children were administered the Children’s Depression Inventory (CDI; Kovacs, 1992) during the laboratory visit to characterize the sample in terms of depression severity. The CDI is a 27-item self-report measure of depressive symptoms in youth aged 7 to 17 years; higher scores on this measure indicate higher depressive symptoms. The CDI has been shown to have good reliability and validity (Stockings et al., 2015).

### Data Analytic Procedure

#### CFAs

We conducted a series of CFAs to examine (a) the latent structure of the SRET (i.e., one- vs. two-factor models) and (b) the fit of different IRT models (i.e., 1 Parameter Logistic [1-PL] vs. 2 Parameter Logistic [2-PL] models). We conducted an initial set of two CFAs to determine whether the SRET shows a one- or two-factor structure. Based on prior theory (Derry & Kuiper, 1981; Hammen & Zupan, 1984), we expected that two factors would emerge, representing children’s positive and negative self-concepts. However, we also tested a one-factor model given the possibility that positive and negative items of the SRET might reflect opposing ends of the same dimension.

We subsequently conducted a second set of four CFAs to compare the fit of two IRT models (i.e., 1-PL and 2-PL models) for each of the positive and negative factors of the SRET. IRT models differ in the number of parameters specified (Birnbaum, 1968). A 2-PL IRT model specifies two parameters: a difficulty parameter (*b*), which refers to the *z*-value along the latent trait distribution at which there is a 50% probability of endorsing both response options, and a discrimination parameter (*a*), which reflects how well an item discriminates between individuals who are low and high on the latent trait. The discrimination parameter additionally reflects the magnitude with which an item is related to the latent trait. Conversely, a 1-PL model specifies only a difficulty parameter, with the discrimination parameter constrained to be zero. We examined both 1-PL and 2-PL models for each of the positive and negative SRET factors to determine the best-fitting model. This permitted not only the evaluation of fit for different IRT models but also provided a test of unidimensionality of the data, a prerequisite for IRT analyses.

We conducted all factor analyses in Mplus Version 8 (Muthén & Muthén, 2017) using a mean- and variance-adjusted weighted least squares estimator (WLSMV), a robust estimator ideal for modeling dichotomous data (Flora & Curran, 2004). WLSMV estimation provides structural equation modeling (SEM) fit indices, permitting evaluations of fit not possible with maximum likelihood (ML) estimation. For the initial two-factor CFA examining latent structure, we used a Geomin rotation as we expected the hypothesized positive and negative factors of the SRET to be moderately correlated based on past work (Dainer-Best et al., 2018; Hayden et al., 2014). The number of random starts was set to 50 for all WLSMV-estimated models.

#### IRT Analyses

After confirming the unidimensionality of both the positive and negative factors of the SRET, we used IRT to explore relationships between children’s latent positive and negative self-concepts and individual SRET items. Specifically, we examined person fit, item fit, item-person fit, conditional reliability, item and test difficulty, and item and test information. IRT analyses were conducted using ML estimation in R using the *mirt* package (Chalmers, 2012). Figures were plotted using the *ggmirt* package for R (Masur, 2022). Data, analysis code, and materials for this study are available upon request from the first author. The present study was not preregistered.

## Results

### Descriptive and Correlational Analyses

Children’s self-reported depressive symptoms fell well below clinical thresholds. The mean score on the CDI was 4.87 (*SD* = 4.99) out of a maximum score of 54. This falls well below clinical thresholds for the CDI (i.e., scores above 17) and is typical for a community sample of preadolescent youth (e.g., Smucker et al., 1986).

Mean positive endorsement scores for our sample were 10.64 (*SD* = 1.84) out of a maximum of 12.00. Mean negative endorsement scores were 1.22 (*SD* = 1.34) out of a maximum of 11.00. WLSMV-derived tetrachoric correlations for study variables are available in Supplemental Appendix A, for both positive (Supplemental Table S1) and negative items (Supplemental Table S2). Inter-item correlations were generally moderate to strong, with nine of these falling above .70 (Supplemental Tables S1–S2). Strong inter-item correlations are to be expected for dichotomous items having high or low endorsement rates. Because the SRET items we examined were necessarily unnuanced toward ensuring that they were developmentally sensitive, many had high (> 90%) or low (< 5%) endorsement rates and endorsement of positive (mean skewness = −3.92) and negative items was skewed (mean skewness = 4.89).^3^ Interestingly, we found significant differences between the U.S. and Canadian samples for endorsement rates of five items (three positive and two negative words). Specifically, Canadian participants endorsed the positive words *lucky*, χ^2^(1) = 6.30, *p* < .05, *popular*, χ^2^(1) = 4.25, *p* < .05, and *terrific*, χ^2^(1) = 6.26, *p* < .05, at significantly higher rates than U.S. participants. Conversely, U.S. participants endorsed the negative words *foolish*, χ^2^(1) = 10.71, *p* < .01, and *lazy*, χ^2^(1) = 5.79, *p* < .05, at significantly higher rates than Canadian participants.^4^

### CFA of One-Factor versus Two-Factor Model

We first conducted a one-factor CFA, wherein positive and negative SRET items reflected opposing ends of a single dimension. We subsequently conducted a two-factor CFA, wherein positive and negative items loaded onto two distinct factors.^5^ As expected, the positive and negative factors were moderately and negatively correlated (*r* = −.40), such that children’s positive self-concepts tended to increase as their negative self-concepts decreased.

Fit indices for the one- and two-factor models are presented in Table 1. The two-factor model showed superior fit to the data on all metrics (i.e., root mean square error of approximation [RMSEA], comparative fit index [CFI], and Tucker–Lewis index [TLI]). We do not report the Standardized Root Mean Square Residual (SRMR) as evidence suggests that this statistic is biased in CFAs with dichotomous variables and should not be interpreted in these cases (Garrido et al., 2016). In addition, we conducted a robust chi-square difference test appropriate for WLSMV estimation (Asparouhov & Muthén, 2006), in which nested models are compared for fit using the DIFFTEST option in Mplus (Kim et al., 2021). This test showed significantly better fit for the two-factor model compared to the one-factor model, χ^2^(1) = 30.43, *p* < .001. Furthermore, fit indices for the two-factor model were superior to those for the one-factor model and approximated conventional benchmarks for good fit (Hu & Bentler, 1999). Thus, we considered the two-factor model the most parsimonious, acceptable model of the SRET and used this to guide subsequent CFA and IRT analyses.

**Table 1:** Fit Statistics for CFA Models.

		χ^2^ (*df*)	*p*	RMSEA [90% CI]	CFI	TLI
*Benchmark for good fit*		Non-significant	>.05	.00–.05	.95–1.00	.95–1.00
*CFA of one-factor vs. two-factor model*						
One-factor model		297.01** (229)	<.01	.03 [.02, .04]	.93	.92
Two-factor model		376.80*** (230)	<.001	.02 [.02, .03]	.95	.94
*CFA of 1-PL vs. 2-PL models*						
Positive item set	1-PL	112.50*** (65)	<.001	.04 [.03, .05]	.95	.95
	2-PL	82.78** (54)	<.01	.03 [.02, .05]	.97	.97
Negative item set	1-PL	75.47* (54)	<.05	.03 [.01, .04]	.95	.94
	2-PL	61.25* (44)	<.05	.03 [.01, .04]	.96	.95

*Note*. RMSEA = root mean square error of approximation [90% confidence interval]; CFI = comparative fit index; TLI = Tucker–Lewis index.

WLSMV estimation used for all CFA models.

We report the standardized WLSMV-derived loadings for the two-factor CFA in Table 2. In general, items loaded as expected onto the positive and negative factors, with all loadings except one, *foolish* (.28), exceeding conventional benchmarks of .40 (Table 2). Notably, *fun* (loading .99) and *ashamed* (loading .97) were the highest loading items on the positive and negative factors, respectively (Table 2).

**Table 2:** Loadings for Two-Factor CFA of SRET Items.

	Positive factor		Negative factor	
Item	Factor loading	*SE*	Factor loading	*SE*
fun	.99	.05		
terrific	.85	.03		
exciting	.83	.04		
proud	.83	.06		
talented	.82	.06		
smart	.80	.06		
strong	.67	.06		
brave	.67	.07		
popular	.61	.04		
friendly^a^	.61	.07		
clever	.58	.09		
lucky	.50	.08		
ashamed^a^			.97	.05
ugly			.83	.07
lonely			.77	.10
boring			.75	.12
angry			.74	.09
stupid			.67	.13
selfish			.61	.07
lazy			.55	.05
sad			.53	.10
clumsy			.50	.05
foolish			.28	.08

*Note.* Factor loadings are standardized probit coefficients due to WLSMV estimation. Probit coefficients represent the change in the *z*-score underlying the latent value of the items. For instance, for a one-unit increase in the latent trait, the *z*-score for the latent score of *terrific* increases by .85. The correlation between the positive and negative factors was −.40.

a Item fit statistics could not be calculated for *friendly* and *ashamed*.

### CFA of 1-PL versus 2-PL Models

Fit statistics for CFAs of positive and negative 1-PL and 2-PL models are reported in Table 1. All models showed relatively large and significant χ^2^ values (Table 1), suggesting poor exact fit. However, exact fit is unlikely for large models such as ours (Maydeu-Olivares & Joe, 2014), which attempt to fit all 2^12^ and 2^11^ possible response patterns in models of positive and negative items, respectively. In these cases, “goodness of approximation” estimates indicating the magnitude of misfit, such as RMSEA, are more useful metrics (Maydeu-Olivares & Joe, 2014). Using these metrics, 1-PL and 2-PL models showed good fit based on conventional benchmarks (Table 1), supporting the unidimensionality of the positive and negative factors. Robust chi-square difference tests further showed that the 2-PL model fit the data significantly better than the 1-PL model for the positive factor, χ^2^(11) = 29.30, *p* < .01, and approached significance for the negative factor, χ^2^(10) = 17.30, *p* = .068.

In summary, initial CFAs indicated that the SRET comprises distinct positive and negative factors. Subsequent CFAs conducted separately for the positive and negative factors confirmed the unidimensionality of both factors, as well as indicated superior fit of 2-PL models compared to 1-PL models. Thus, we hereafter focus on 2-PL models of positive and negative SRET items in IRT analyses. However, we provide results for 1-PL models in Supplementary Appendix C.

### IRT Analyses

Several assumptions underlie IRT modeling, including unidimensionality, local independence, and monotonicity (Nguyen et al., 2014). We provide details on checks of these assumptions in Supplementary Appendix D. To summarize, CFAs detailed above support the unidimensionality of the positive and negative factors of the SRET. In addition, we found no violations of monotonicity for the positive or negative SRET factors (Supplementary Appendix D). Finally, we found few issues of local dependence across models, although inspection of residual correlation matrices suggested that the positive items *smart* and *fun* and the negative item *lonely* may be redundant with other items (Supplementary Appendix D). Removing these items from the models of positive and negative items, respectively, produced minimal changes in model fit and parameters.^6^ In the interest of providing IRT metrics for as many items as possible, we retained these items in subsequent analyses.

#### Evaluations of Fit for 2-PL Models

##### Person Fit

Person-fit statistics reflect the extent to which models fit individuals and can detect outliers and aberrant response behavior, such as misunderstanding items or inattentive responding. Visual inspection of person-fit plots and review of person-fit infit and outfit statistics showed that the percentage of children with infit and outfit *z*-scores falling outside the range of −1.96 and +1.96 was less than 1% for the model of the positive item set and less than 2% for the model of the negative item set. Thus, the vast majority of individual response patterns fit the models well, and we retained all participants in subsequent analyses.

##### Item fit

While overall fit statistics are useful summary statistics, reviewing fit statistics for individual items is important for pinpointing specific areas of misfit. We reviewed Pearson’s χ^2^ (*S*-χ^2^) statistics (Orlando & Thissen, 2000) as well as infit and outfit statistics. All items showed good fit in 2-PL models, with the exception of the positive item *friendly* and negative item *ashamed*, for which fit statistics could not be computed (Table 3).

**Table 3:** Parameter Estimates for 2-PL Models.

Positive item set						Negative item set					
Item	*S*-χ^2^ (*df*)	*p*	RMSEA	*b*	*a*	Item	*S*-χ^2^ (*df*)	*p*	RMSEA	*b*	*a*
*Benchmark for good fit*	Non-significance	>.05	.00–.05	–	–	*Benchmark for good fit*	Non-significance	>.05	.00–.05	–	–
fun	1.85 (1)	.174	.04	−2.60	2.81	ashamed^a^	–	–	–	2.38	26.40
terrific	7.02 (5)	.219	.03	−1.39	2.20	ugly	2.98 (4)	.561	.00	3.26	1.37
exciting	4.52 (5)	.478	.00	−1.85	2.05	lonely	4.81 (3)	.186	.04	2.57	2.12
proud	5.51 (6)	.481	.00	−2.07	2.08	boring	2.89 (3)	.410	.00	3.04	1.63
talented	8.49 (6)	.204	.03	−1.98	2.00	angry	2.47 (3)	.481	.00	2.17	2.38
smart	3.59 (6)	.733	.00	−2.19	2.49	stupid	1.10 (3)	.778	.00	2.87	1.98
strong	6.79 (5)	.237	.03	−1.35	1.78	selfish	3.64 (3)	.304	.02	2.37	2.06
brave	5.99 (5)	.307	.02	−1.67	1.50	lazy	4.89 (2)	.087	.05	.60	1.39
popular	1.94 (3)	.586	.00	−.38	1.51	sad	.30 (4)	.990	.00	2.71	1.41
friendly^a^	–	–	–	−3.65	1.85	clumsy	1.43 (2)	.490	.00	.20	1.45
clever	1.55 (7)	.980	.00	−2.42	1.27	foolish	4.74 (3)	.192	.03	1.63	1.10
lucky	9.50 (7)	.219	.03	−1.92	1.00						

Note. *S*-χ2 = Pearson’s chi-square; RMSEA = root mean square error of approximation; *b* = difficulty parameter; *a* = discrimination parameter.^a^Item fit statistics could not be calculated for *friendly* and *ashamed*.

* *p* < .05. **p < .01. ***p < .001.

We further reviewed infit and outfit statistics for each item. These chi-square statistics measure the degree of fit of individual items compared to the average of other items in a scale, with values under 0.5 indicating underfit and values over 1.5 indicating overfit (Linacre, 2002). Values outside the range of 0.5 to 1.5 are less productive for measurement, although only values above 1.5 are degrading to the model and threaten its validity (Linacre, 2002). Infit down-weights outliers and is more sensitive to unexpected responses on items that are roughly targeted to a person’s position on the latent trait. Outfit is sensitive to outliers, including unexpected responses for a person given their position on the latent trait.

**Figure 1.**
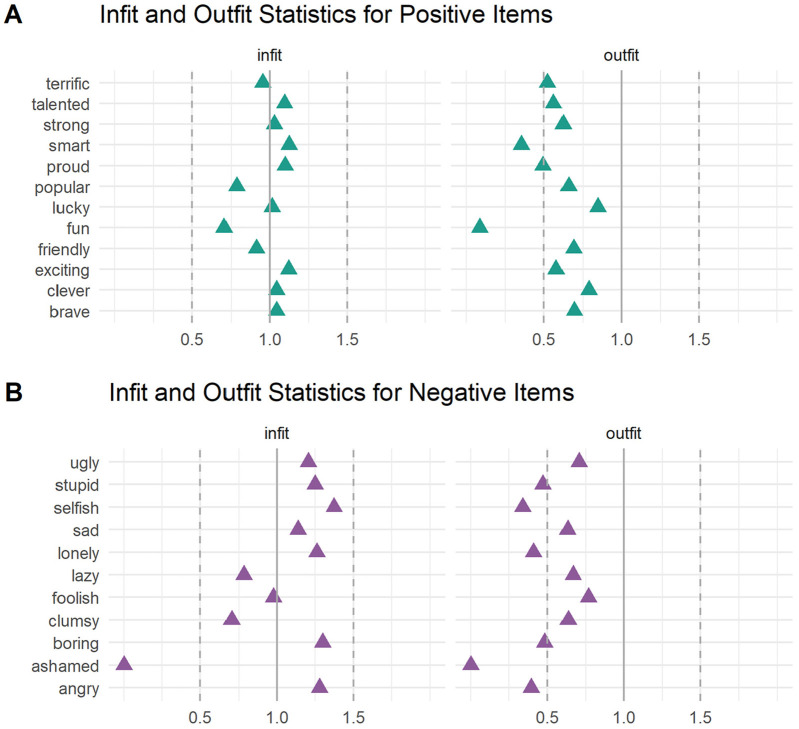
Item Infit and Outfit Statistics. *Note.* Items with values within 0.5 and 1.5 are considered to be productive for measurement.

Infit was excellent for the 2-PL model for the positive item set, with all items falling within the ideal range of 0.5 and 1.5 (Figure 1A). No items showed outfit above 1.5, although three items (*smart*, *proud*, and *fun*) fell below 0.5 (Figure 1A). Low outfit values do not threaten the validity of the model, but rather suggest model overfit, such that responses to these items have low variance (e.g., are endorsed by virtually all respondents) and are therefore less useful for measuring children’s positive self-concept. This was unsurprising given that endorsement rates exceeded 93% for these three items.

We found a similar pattern of infit and outfit for the 2-PL model for the negative item set, although a greater number of negative items showed low outfit. All but one item (*ashamed*) fell within the ideal range for infit (Figure 1B). No items showed outfit above 1.5, though six items (*stupid, selfish, lonely, boring, ashamed*, and *angry*) fell below 0.5, suggesting that these items had such low variance (i.e., endorsement rates less than 5%) as to be too “predictable” and therefore less useful for measuring children’s negative self-concept (Figure 1B). Together, these findings suggest that the negative item *ashamed* shows particularly low measurement utility. Considered alongside the poor fit statistics for this item (Table 3), this prompted us to exclude the item *ashamed* from our subsequent figures, as doing so preserved interpretation of the other negative items.

##### Item-person fit

**Figure 2.**
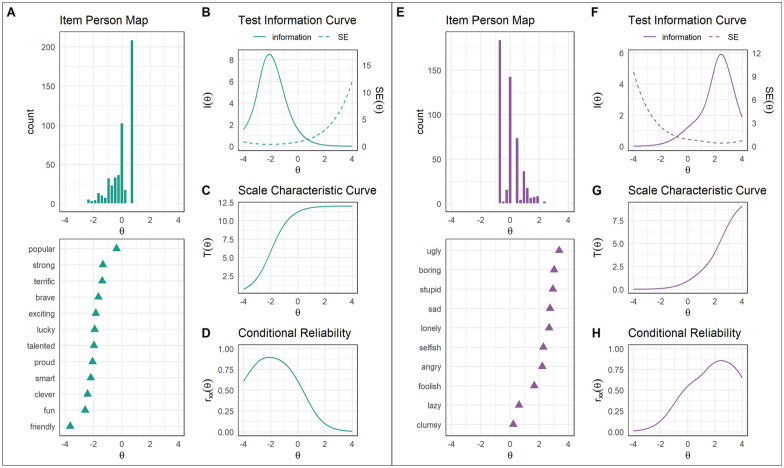
Summary Statistics for 2-PL Models of Positive and Negative Items. *Note. SE* = standard error. *r*_xx_ = conditional reliability. Theta (θ) = children’s underlying positive self-concept. (A–D) 2-PL model for positive item set. (E–H) 2-PL model for negative item set. The item *ashamed* was not included due to its poor fit and large *a* parameter, which distorted visualizations of summary statistics for the overall scale.

We present item-person (or Wright) maps for the models of the positive item set (Figure 2A) and negative item set (Figure 2E). These maps depict how well items cover the range of the latent trait by comparing the distribution of the latent trait against item difficulty (*b* parameter) using the same scale. Items should ideally span the full range of the latent trait exhibited by the sample. The latent trait distribution for the model of the positive item set was negatively skewed, such that only a small proportion of children possessed low positive self-concepts (Figure 2A). Because the threshold for endorsement of most positive words occurred along the left tail of the person distribution, positive words tended to map the portion of the sample exhibiting particularly low positive self-concept and appeared less well suited to mapping the full range of self-concept present in the sample (Figure 2A).

The reverse was true for the model of the negative item set. Latent trait distributions for this model were positively skewed, such that only a small proportion of children exhibited very negative self-concepts (Figure 2E). In contrast to the model of the positive item set, the threshold for endorsement of most negative words occurred along the right tail of the person distribution, indicating that negative items best mapped the portion of the sample exhibiting very negative self-concept (Figure 2E).

##### Scale Characteristic Curves

The scale characteristic curve (SCC) depicts the relationship between theta (i.e., children’s latent positive or negative self-concept) and the number-endorsed score. It shows the extent to which the total number of positive (or negative) items endorsed is a sufficient estimation of children’s positive (or negative) self-concept. SCCs are plotted for the 2-PL model for the positive item set in Figure 2C. The number of items endorsed increased as theta values became less negative before plateauing at theta values over 1.0. This suggests that the number-endorsed score is a sufficient estimation of children’s positive self-concepts when positive self-concepts are in the below-average to average range.

SCCs are plotted for the 2-PL model for the negative item set in Figure 2G. The SCC for this model showed the opposite pattern to the model of the positive item set. The number of items endorsed increased as theta values became more positive before plateauing at theta values below −1.0, suggesting that the number-endorsed score estimates children’s negative self-concepts well when these are in the average to above-average range.

##### Conditional Reliability

Conditional reliability is the reliability of a scale at different levels of theta. This stands in contrast to classical test theory, which assumes that reliability is constant across the latent trait. Using a cut-off of .75, conditional reliability was best in the −3.0 ≤θ≤−0.5 range for the 2-PL model for the positive item set (Figure 2D). In contrast, conditional reliability was best in the 1.5 ≤θ≤ 3.5 range for the 2-PL model for the negative item set (Figure 2H). Because conditional reliability is mathematically related to the test information and conditional standard error functions, reliability was accordingly poor at higher values of theta in the model of the positive item set and lower values of theta in the model of the negative item set.

#### Difficulty and Discrimination Parameters

##### Difficulty Parameters

We report the difficulty parameters (*b*) for positive and negative SRET items in Table 3. Difficulty parameters reflect the position along the latent trait distribution where an individual is equally likely to endorse or not endorse an item. In other words, this value is the threshold at which the probability of endorsing an SRET word flips between probable and improbable. For example, the 2-PL difficulty parameter for the positive word *strong* (−1.35) indicates that a child scoring lower than 1.35 standard deviations below the mean positive self-concept is unlikely to endorse *strong* as self-descriptive. Conversely, the 2-PL difficulty parameter for the negative word *lazy* (.60) indicates that a child scoring lower than .60 standard deviations above the mean negative self-concept is unlikely to endorse *lazy*. Difficulty parameters can be seen in item characteristic curves (ICCs) as the 50% endorsement rate and in item information curves (IICs) as the location of peak information.

**Figure 3.**
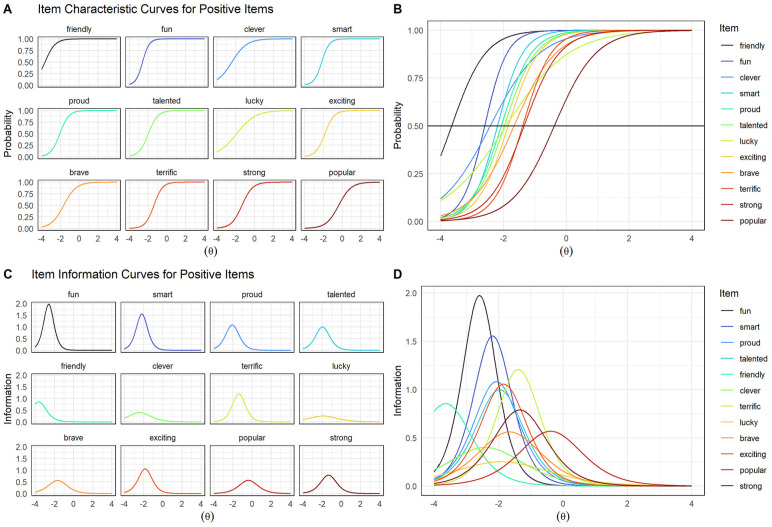
Item Characteristic and Information Curves for 2-PL Model of Positive Item Set. *Note.* Theta (θ) = Children’s latent positive self-concept. Item characteristic curves (ICCs) shown plotted individually (A) and together (B). Item information curves (IICs) shown plotted individually (C) and together (D). ICCs display the probability of a child endorsing an item as a function of overall positive or negative self-concept (theta). For instance, the probability that a child possessing an average positive self-concept would describe herself as *lucky* is approximately 88% (B). IICs are the mathematical first derivatives of ICCs and display the precision with which an item estimates scores on the latent trait. The inflection point of an ICC aligns with the peak of its IIC.

Difficulty parameters for all positive items were negative, such that most children tended to endorse positive items (Figure 3B). This pattern was observed in the extreme for items *friendly*, *fun*, and *clever*; these words went unendorsed only by children with the lowest positive self-concepts. Accordingly, the majority of positive words provided the most information at very low values of theta (i.e., two standard deviations below the mean; Figure 3D). Thus, these words appear well suited to assessing children with average or lower positive self-concepts, but less useful for measurement of children with above-average or higher positive self-concepts. The exception to this pattern was the item *popular*, for which the inflection point (−.38) and information curve fell closer to theta zero. Compared to other positive items, *popular* better mapped the average range of positive self-concept—an important range given that this is where most children are likely to fall (Figure 3). However, a majority of children nevertheless endorsed this most “difficult” word (*popular*).

**Figure 4.**
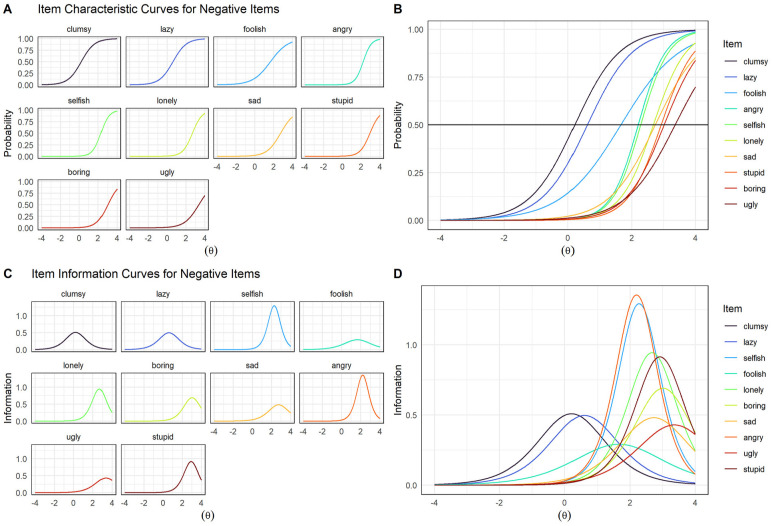
Item Characteristic and Information Curves for 2-PL Model of Negative Item Set. *Note.* Theta (θ) = Children’s latent negative self-concept. Item characteristic curves shown plotted individually (A) and together (B). Item information curves shown plotted individually (C) and together (D). ICCs display the probability of a child endorsing an item as a function of overall positive or negative self-concept (theta). For instance, the probability that a child possessing an average negative self-concept would describe herself as *lazy* is approximately 30% (D). IICs are the mathematical first derivatives of ICCs and display the precision with which an item estimates scores on the latent trait. The inflection point of an ICC aligns with the peak of its IIC.

ICCs and IICs for the 2-PL model for the negative item set are shown in Figure 4. In contrast to positive items, most negative items went unendorsed by children, as indicated by very positive *b* values (Figure 4B). For example, even for children falling two standard deviations above the mean negative self-concept, the probability of endorsing *ugly*, *stupid*, or *selfish* was less than 10%. The item *clumsy* had the highest probability of endorsement (barring *ashamed*, which was excluded due to its very high *a* value [26.40], which obscured meaningful differences between other items when plotted). The item *stupid* had the highest probability of endorsement at theta values up to 2.0, though *ugly* had the highest probability for theta values greater than 2.0. In summary, most negative items appear best equipped for assessing children with highly negative self-concepts.

##### Discrimination Parameters

Discrimination parameters (*a*) reflect how well an item distinguishes between individuals with different levels of the latent trait. They also reflect the magnitude to which items are related to the latent trait. Larger *a* values indicate better measurement of the latent trait and better ability to differentiate between individuals across the latent trait. Discrimination parameters influence the slopes of ICCs and the amplitudes of IICs. In 2-PL models, where the discrimination parameter is allowed to vary across items, higher *a* values produce steeper ICCs and taller IICs.

Items varied widely in the extent to which they provided information about children’s positive self-concepts. Notably, when taking into account the ability of items to differentiate between individuals, items *lucky* and *clever* showed lower measurement utility (Figure 3D). Specifically, these items showed the smallest IICs as well as gentler ICC slopes compared to other items covering the same ranges of theta, such as *fun* and *smart* (Figure 3). Thus, *lucky* and *clever* provided the least information about children’s positive self-concepts and were less efficient than other items at differentiating between individuals across the latent trait. In contrast, items *fun* and *smart* provided the most information, although at very negative values of theta (i.e., below −2.0; Figure 3D); thus, these items were efficient at differentiating between individuals falling at different positions on the latent trait, but only for children with very low positive self-concepts (Figure 3). Items *proud*, *talented*, and *exciting* showed highly similar ICCs and IICs, suggesting possible redundancy between these items (Figure 3).

Negative items tended to provide the most information at positive values of theta (Figure 4D). Thus, most negative words appeared excellent for differentiating between children with very negative self-concepts (i.e., two standard deviations above the mean), but less useful for assessing children whose negative self-concepts fell within the average range or below. Exceptions to this pattern were *clumsy*, *lazy*, and *foolish*, for which IICs spanned both negative and positive values of theta (Figure 4D), suggesting that these words are better able to differentiate between children across a broader range of negative self-concept severity.

##### Test Information Curve

The test information curve (TIC) is a summary statistic of how well items of a scale collectively provide statistical information about the latent trait. These plots are enhanced by corresponding conditional standard error curves, which depict the precision with which scores can be estimated across different values of theta. The TIC for the 2-PL model of the positive SRET scale is shown in Figure 2B. This figure indicates that the positive SRET scale performs well when estimating negative scores, but has less precision when estimating theta scores greater than zero. Specifically, the range of theta scores for which the positive scale showed good estimation was −4.0 ≤θ≤ 0.5 (Figure 2B). Thus, the positive scale shows good estimation for positive self-concepts that fall below average, but performs poorly when they are above average.

The TIC for the 2-PL model of the negative SRET scale is shown in Figure 2F. In contrast to the positive scale, the negative scale adequately estimates positive values of theta, but falters when estimating theta scores below zero. Specifically, the range of theta scores for which the negative scale showed good estimation was −0.5 ≤θ≤ 4.0 (Figure 2F). Thus, in contrast to the positive scale, the negative scale shows good estimation for high negative self-concepts and poor estimation for low negative self-concepts.

## Discussion

Investigating individual differences in cognitive vulnerability in youth is critical for elucidating processes of depression risk across development. The SRET indexes biases involved in processing self-referent information. Despite decades of use, however, no study has yet examined the structural validity and item functioning of this measure. We therefore examined the factor structure and item performance of a developmentally sensitive version of the SRET using CFA and IRT in a large community sample of preadolescent youth. Consistent with hypotheses, the SRET exhibited a two-factor structure, comprising positive and negative factors. We found good fit for most items in CFA and IRT models. The 2-PL IRT models showed superior fit to the 1-PL models. Positive items were endorsed by most children, and best estimated information about positive self-concepts below average levels of positivity. Conversely, negative items were unendorsed by most children, and best estimated information about negative self-concepts above average levels of negativity. Thus, items showed greatest utility for assessing children’s positive and negative self-concepts when these were particularly low and high, respectively. Based on our findings, we provide recommendations for future implementations of the SRET with youth.

As would be expected in a community sample, children tended to endorse most positive words and not endorse most negative words. Indeed, for most negative words in our study, the endorsement rate for a child with an average negative self-concept was less than 5%. In contrast, for most positive words, the endorsement rate for a child with an average positive self-concept was over 95%. These findings indicate a self-positivity bias similar to that reported by others using the SRET with preadolescent children (Hudson et al., 2021; P. Liu & Tan, 2023; Timbremont & Braet, 2004). Evidence suggests that this bias is present throughout childhood until early adolescence, at which time self-concepts become more differentiated and less positive and/or more negative overall (Crone et al., 2022; Harter, 2012).

Our findings are constrained by the limitations of children’s cognitive ability and reading level. That is, SRET words were selected for Grade 3 readability to limit the effects of children’s verbal ability on word endorsement. As a result, items were less nuanced in their connotations (e.g., *ugly*, *stupid*, and *angry* instead of *plain*, *naïve*, and *argumentative*). Children may have been less likely to endorse unambiguously negative words as self-descriptive, as well as more likely to endorse unambiguously positive words. Thus, when using the SRET with preadolescent children, the inclusion of items that balance readability and nuance, such as *popular* and *clumsy*, is critical. Of all items examined, these best mapped ranges of the latent distribution where most typically developing children are expected to fall (i.e., above-average positive self-concepts and below-average negative self-concepts). Thus, these items should be retained in future implementations of the SRET with preadolescent children. In addition, while pragmatic considerations prevented the sourcing and validation of new SRET words in the present study, we encourage development of additional nuanced SRET items for use with children.

While *popular* and *clumsy* appeared particularly useful, other items performed poorly in our models and/or added little to the overall utility of the SRET and may therefore be candidates for removal. Ultimately, the decision to discard items should be made after considering overall and item fit statistics, coverage of the latent construct, and item content (Fährmann et al., 2022). With respect to positive items, the item *friendly* performed poorly in several of these domains; specifically, fit statistics were unable to be computed for this item in the positive IRT model and it mapped only a small portion of the latent construct (i.e., it had very low difficulty and discrimination parameters, such that all but three children endorsed this word). In summary, we advise against including *friendly* in future implementations of the SRET with preadolescent children. Researchers may also wish to consider the utility and practical significance of the items *fun, smart*, and *lucky* when using the SRET with community samples of preadolescent children. However, we recommend continued use of the following positive SRET items: *terrific, exciting, proud, talented, strong, brave, popular*, and *clever*.

Several negative items also performed poorly in our models. Notably, *ashamed* showed poor fit on most metrics and low variance. It is possible that our participants had difficulty understanding this word, which is more abstract than our other words; however, all children in our sample were well beyond the mean age of acquisition (7.11 years; Kuperman et al., 2012). Given these shortcomings, we advise against including *ashamed* in future implementations of the SRET with preadolescent children. Similarly, *foolish* showed poor fit according to some metrics and good fit according to others. However, this item mapped the average range of the distribution of children’s negative self-concepts in our community sample better than most negative words, possibly because of its milder negativity. Researchers may therefore wish to retain *foolish* in cases where sufficient coverage of the latent construct is of greater theoretical significance than statistical fit, particularly in large samples where retaining small numbers of misfitting items is unlikely to bias the results of IRT models (Fährmann et al., 2022). In summary, we advise against including the item *ashamed*, and researchers should consider the utility and practical significance of the item *foolish* in future implementations of the SRET with community samples of preadolescent children. However, we recommend continued use of the following negative SRET items: *ugly, lonely, boring, angry, stupid, selfish, lazy, sad*, and *clumsy.*

### Limitations

There are several limitations to our study that deserve mention. First, although the demographic makeup of the sample was representative of the geographic areas from which participants were recruited, the homogeneity of the sample limits the generalizability of our findings to more diverse child populations, particularly racial/ethnic minority groups. Similarly, our findings may not generalize to children experiencing clinically significant depressive symptoms. However, evidence suggests that cognitive vulnerability to depression in late childhood exists along a spectrum (R. T. Liu et al., 2019). Thus, we hypothesize that items may in fact show particularly good utility with depressed youth, as these children would be expected to fall within the ranges of positive and negative self-concept well mapped by the items we examined (i.e., highly negative self-concepts and very low positive self-concepts). Nevertheless, investigating the measurement utility of SRET items in diverse and clinical child populations remain an important avenue for future research.

Second, we acknowledge the possibility that differences in the method of SRET administration—handheld flash cards (U.S. sample) versus computer software (Canadian sample)—may have influenced children’s responses to word stimuli. For instance, it is possible that the presence of the examiner may have deterred U.S. participants from endorsing negative SRET words. However, we note that U.S. participants endorsed certain negative items at significantly higher rates than Canadian participants; given the general similarity of the two samples, this suggests that the method of administration did not diminish children’s willingness to endorse negative self-content.

Lastly, analyses of measurement invariance and differential item functioning across sex and developmental stages are needed to speak to the measurement utility of SRET items in subpopulations of youth. We focused on children in late childhood, a critical developmental period because it precedes increases in depression risk that occur during adolescence (Thapar et al., 2012). However, youth may interpret SRET words differently depending on their developmental stage. For instance, preadolescent children tend to use temporal comparisons to define their self-concept (e.g., perceiving oneself as smarter than one’s past self) whereas adolescents tend to use social comparisons (e.g., perceiving oneself as smarter than other people) (Guerin & Tatlow-Golden, 2019; Harter, 2012). Relatedly, establishing measurement invariance across boys and girls is an important prerequisite for using the SRET to investigate sex differences in children’s self-concepts. For example, prior work suggests that girls rate themselves more positively in academic domains of self-concept than boys (Van der Aar et al., 2018). However, whether similar findings in studies using the SRET reflect a meaningful sex difference or simply differences in the ways items function for boys versus girls is unknown. In conclusion, while examining differential item functioning across children’s sex and age was beyond the scope of the present study, it is nevertheless an important area for future research.

## Supplemental Material

sj-docx-1-asm-10.1177_10731911241289249 – Supplemental material for Latent Structure and Item Functioning of Self-Referent Encoding Task Word Stimuli in Preadolescent Youth Supplemental material, sj-docx-1-asm-10.1177_10731911241289249 for Latent Structure and Item Functioning of Self-Referent Encoding Task Word Stimuli in Preadolescent Youth by Lindsay N. Gabel, Thomas M. Olino, Brandon L. Goldstein, Daniel N. Klein, Kasey Stanton and Elizabeth P. Hayden in Assessment

## Notes

1. There were minimal changes to the results of CFA and IRT analyses when using only the U.S. sample versus using the combined U.S. and Canadian sample; however, these are available upon request from the first author.

2. The negative mood induction is considered an essential component of the SRET, particularly in community samples, because it activates latent cognitive vulnerability to depression (Taylor & Ingram, 1990). In this way, the negative mood induction is thought to potentiate differences in SRET indices between youth with varying cognitive vulnerabilities to depression that could not otherwise be captured.

3. In this way, the relatively unnuanced nature of the binary items likely contributed to our high inter-item correlations. The high correlations between items that are semantically unrelated (e.g., *lazy* and *selfish*, *r* = .78) support this possibility. At the same time, high inter-item correlations may point to redundancy among items. We note that the items *fun*, *ashamed*, and *smart* accounted for nearly all inter-item correlations above .70 (except *lazy-selfish* noted above), suggesting that these three items may be redundant with other items.

4. We examined the impact of differential item responding across samples via tests of measurement invariance (See Supplementary Appendix B for details). These analyses supported scalar invariance of the SRET across samples, providing justification for combining the U.S. and Canadian samples for IRT analyses. In summary, differential responding across samples for five SRET items likely had limited impact on our results.

5. The four neutral SRET items were not included in CFAs, as their purpose was solely to reduce primacy and recency effects.

6. Specifically, fit of the 2-PL model for the positive item set was excellent regardless of whether these three items were included, χ^2^(54) = 82.78, *p* < .01; RMSEA = .03, CFI = .97, TLI = .97, or excluded, χ^2^(35) = 30.69, *p* = .68; RMSEA = .00, CFI = 1.00, TLI = 1.00. Similarly, fit of the 2-PL model for the negative item set was comparable when including, χ^2^(44) = 61.25, *p* < .05; RMSEA = .03, CFI = .96, TLI = .95, and excluding, χ^2^(35) = 47.76, *p* = .07; RMSEA = .03, CFI = .96, TLI = .95, the three items. In addition, IRT parameters showed relatively little change when excluding these three items, with the maximum change in item difficulty and discrimination parameter estimates being .36 and .21 units, respectively, across all items (except for *ashamed*, which ultimately proved to be a poorly fitting item and outlier).
