# Supplementary material for: Latent Structure and Item Functioning of Self-Referent Encoding Task Word Stimuli in Preadolescent Youth
Source: Assessment. 2024 Nov 23;32(7):1103–19. doi: 10.1177/10731911241289249 (PMC12397560; doi:10.1177/10731911241289249)
Supplement: sj-docx-1-asm-10.1177_10731911241289249 – Supplemental material for Latent Structure and Item Functioning of Self-Referent Encoding Task Word Stimuli in Preadolescent Youth [file sj-docx-1-asm-10.1177_10731911241289249.docx]

**Appendix A**

**Inter-Item Correlations**

**Table S1**

*Inter-Item Correlations for Demographic and Positive SRET Items*

|  | 1 | 2 | 3 | 4 | 5 | 6 | 7 | 8 | 9 | 10 | 11 | 12 | 13 | 14 |
| --- | --- | --- | --- | --- | --- | --- | --- | --- | --- | --- | --- | --- | --- | --- |
| 1. Sex | – |  |  |  |  |  |  |  |  |  |  |  |  |  |
| 2. Age | .03 | – |  |  |  |  |  |  |  |  |  |  |  |  |
| 3. PPVT | .03 | -.15 | – |  |  |  |  |  |  |  |  |  |  |  |
| 4. fun | .10 | .09 | -.04 | – |  |  |  |  |  |  |  |  |  |  |
| 5. terrific | .00 | .15 | -.05 | .75 | – |  |  |  |  |  |  |  |  |  |
| 6. exciting | .25 | .09 | -.14 | .84 | .63 | – |  |  |  |  |  |  |  |  |
| 7. proud | -.08 | .07 | -.10 | .62 | .68 | .53 | – |  |  |  |  |  |  |  |
| 8. talented | .15 | .14 | .05 | .68 | .61 | .50 | .55 | – |  |  |  |  |  |  |
| 9. smart | -.09 | -.06 | .07 | .60 | .69 | .49 | .63 | .77 | – |  |  |  |  |  |
| 10. strong | .08 | .09 | -.08 | .39 | .57 | .56 | .45 | .44 | .50 | – |  |  |  |  |
| 11. brave | .09 | .03 | -.10 | .31 | .50 | .60 | .43 | .51 | .27 | .62 | – |  |  |  |
| 12. popular | -.10 | .15 | -.09 | .60 | .49 | .48 | .50 | .57 | .54 | .55 | .38 | – |  |  |
| 13. friendly | -.18 | .09 | -.45 | .64 | .51 | .62 | .41 | .38 | .25 | .48 | .52 | .43 | – |  |
| 14. clever | -.07 | -.02 | .07 | .57 | .43 | .38 | .42 | .37 | .78 | .42 | .23 | .34 | .36 | – |
| 15. lucky | .10 | -.13 | .09 | .12 | .38 | .39 | .51 | .29 | .34 | .38 | .47 | .31 | .20 | .34 |

*Note*. PPVT = Peabody Picture Vocabulary Test scores at age 3. For sex, 0 = male, 1 = female. Correlations with age and PPVT scores are point-biserial correlations; all other correlations are tetrachoric correlations.

**Table S2**

*Inter-Item Correlations for Demographic and Negative SRET Items*

|  | 1 | 2 | 3 | 4 | 5 | 6 | 7 | 8 | 9 | 10 | 11 | 12 | 13 |
| --- | --- | --- | --- | --- | --- | --- | --- | --- | --- | --- | --- | --- | --- |
| 1. Sex | – |  |  |  |  |  |  |  |  |  |  |  |  |
| 2. Age | .03 | – |  |  |  |  |  |  |  |  |  |  |  |
| 3. PPVT | .03 | -.15 | – |  |  |  |  |  |  |  |  |  |  |
| 4. ashamed | .03 | -.25 | .23 | – |  |  |  |  |  |  |  |  |  |
| 5. ugly | .16 | -.13 | .00 | .71 | – |  |  |  |  |  |  |  |  |
| 6. lonely | .33 | -.04 | .12 | .85 | .67 | – |  |  |  |  |  |  |  |
| 7. boring | -.02 | .00 | -.17 | .52 | .48 | .28 | – |  |  |  |  |  |  |
| 8. angry | -.29 | -.04 | -.16 | .79 | .14 | .56 | .18 | – |  |  |  |  |  |
| 9. stupid | .09 | .30 | .04 | .77 | .52 | .52 | .35 | .42 | – |  |  |  |  |
| 10. selfish | -.18 | .21 | .09 | .42 | .35 | .35 | .39 | .62 | .24 | – |  |  |  |
| 11. lazy | .00 | .08 | -.02 | .51 | .29 | .45 | .50 | .53 | .44 | .78 | – |  |  |
| 12. sad | .19 | -.04 | -.07 | .61 | .44 | .43 | .47 | .49 | .19 | .33 | .13 | – |  |
| 13. clumsy | .30 | .02 | .05 | .43 | .10 | .36 | .41 | .58 | .49 | .40 | .37 | .48 | – |
| 14. foolish | -.02 | .17 | -.02 | .53 | .17 | .17 | .23 | .28 | .51 | .26 | .35 | .33 | .44 |

*Note*. PPVT = Peabody Picture Vocabulary Test scores at age 3. For sex, 0 = male, 1 = female. Correlations with age and PPVT scores are point-biserial correlations; all other correlations are tetrachoric correlations.

**Appendix B**

**Measurement Invariance Across US and Canadian Samples**

We examined the impact of differential item responding across samples by examining results when using (1) only the US sample (*N* = 430), (2) only the Canadian sample (*N* = 78), and (3) the combined US and Canadian sample (*N* = 508). We performed separate CFAs with each of these samples and found good fit for a two-factor model in all. Indeed, fit indices were comparable across the US sample (*χ^2^*(229) = 299.79, *p* < .01; RMSEA = .03; CFI = .96; TLI = .95), Canadian sample (*χ^2^*(169) = 209.14, *p* < .05; RMSEA = .06; CFI = .94; TLI = .93), and combined sample (*χ^2^*(230) = 376.80, *p* < .001; RMSEA = .02; CFI = .95; TLI = .94).

Having found acceptable fit for a two-factor structure in each sample, we subsequently performed a multi-group CFA to test measurement invariance across the US and Canadian samples. This analysis was exploratory given the small size of the Canadian sample. It also required the removal of three items which showed no variance in the Canadian sample (*selfish*, *stupid*, and *friendly*). Results of invariance testing showed excellent fit for the configural model (*χ^2^*(338) = 424.66, *p* < .001; RMSEA = .03; CFI = .96; TLI = .95) as well as the scalar model (*χ^2^*(354) = 437.91, *p* < .01; RMSEA = .03; CFI = .96; TLI = .95). We note that the metric model is not independently testable when binary variables are used as estimation of loadings and thresholds are dependent. Importantly, there was no significant difference in fit between the configural and scalar models (*χ^2^*(16) = 14.55, *p* = .58). These findings support the scalar invariance of the SRET across the US and Canadian samples and provide justification for combining the two samples for IRT analyses.

**Appendix C**

**Results of 1 Parameter Logistic (1-PL) Models**

***Rationale for Testing 1-PL Models***

Within measurement theory, there are two scholarly traditions with diverging philosophical underpinnings. Proponents of the Rasch approach to measurement prioritize the development of an objective measure whose item parameters are invariant across samples (Stemler & Naples, 2021). Applied to the SRET, the Rasch model assumes that SRET words function the same way for all children, regardless of how positive or negative their self-concept, such that children’s self-concepts are sufficiently estimated by the total number of positive/negative words they endorse. Thus, using the Rasch approach permits conclusions about the universal measurement utility of SRET items. In contrast, proponents of the IRT approach prioritize the derivation of latent trait estimates for a particular sample (Stemler & Naples, 2021). Applied to the SRET, the IRT model assumes that SRET words function differently for children with higher or lower positive/negative self-concepts. In this way, children’s self-concepts are determined not only by the number of positive/negative words they endorse, but also by *which* words they endorse. In summary, the Rasch approach prioritizes the fit of the data to the model, yielding measurement models, while the IRT approach prioritizes the fit of the model to the data, yielding statistical models.

Rasch and IRT models differ in the number of parameters specified (Birnbaum, 1968). The Rasch model is mathematically equivalent to a 1-PL IRT model (Stemler & Naples, 2021).^[[1]](#footnote-1)^ Unlike 2-PL models, 1-PL models speak to the sample-independent measurement utility of SRET items. We therefore examined 1-PL models of the SRET alongside 2-PL models to provide researchers with rich information with which to evaluate the measurement utility of SRET items.

***Results***

**Item fit.** For the 1-PL model for the positive item set, the items *fun* and *lucky* showed poor fit, as indicated by significant *S*-χ^2^ values and RMSEAs above 0.5 (Table S3). As in the 2-PL model, fit statistics for the item *friendly* were unable to be computed, possibly because of the very high endorsement rate for this word (Table S3). For the 1-PL model for the negative item set, items *ashamed* and *foolish* demonstrated misfit (Table S4).

We further reviewed infit and outfit statistics for each item. Infit was excellent for the 1-PL model for the positive item set, with all items falling within the ideal range of 0.5 and 1.5 (Figure B1A). No items showed outfit above 1.5, though three items (*smart*, *proud*, and *fun*) fell below 0.5, as in the 2-PL model (Figure B1A).

**Figure B1**

*Item Infit and Outfit Statistics*


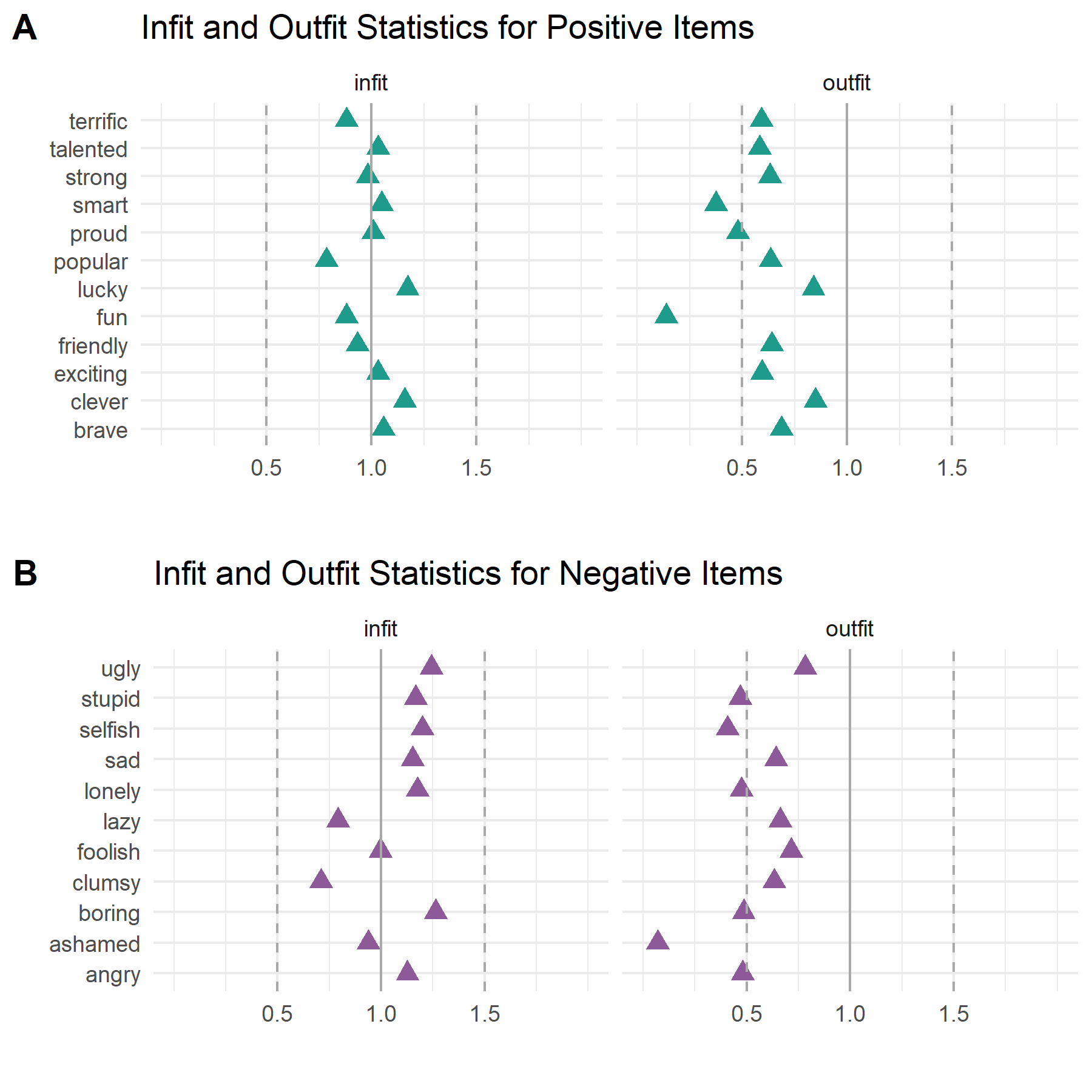


*Note.* Items with values within 0.5 and 1.5 are considered to be productive for measurement.

We found a similar pattern of infit and outfit for the 1-PL model for the negative item set, though a greater number of negative items showed low outfit. All but one item (*ashamed*) fell within the ideal range for infit (Figure B1B). No items showed outfit above 1.5, though six items (*stupid, selfish, lonely, boring, ashamed*, and *angry*) fell below 0.5, suggesting that these items had such low variance (i.e., endorsement rates less than 5%) as to be too “predictable” and therefore less useful for measuring children’s negative self-concept (Figure B1B). As with the negative 2-PL model, we excluded the item *ashamed* from our subsequent figures for the 1-PL model, as doing so preserved interpretation of the other negative items.

**Item-person fit.** The latent trait distribution for the1-PL model or the positive item set negatively skewed, such that only a small proportion of children possessed low positive self-concepts (Figure B2A). Because the threshold for endorsement of most positive words occurred along the left tail of the person distribution, positive words tended to map the portion of the sample exhibiting particularly low positive self-concept and appeared less well suited to mapping the full range of self-concept present in the sample (Figure B2A). As in the positive 2-PL model, the item *popular* overlapped well with the person distribution (Figure B2A).

The reverse was true for the model of negative items. Latent trait distributions for this model were positively skewed, such that only a small proportion of children exhibited very negative self-concepts (Figure B1E). In contrast to the model of positive items, the threshold for endorsement of most negative words occurred along the right tail of the person distribution, indicating that negative items best mapped the portion of the sample exhibiting very negative self-concept (Figure B1E).

**Figure B2**

*Summary Statistics for 1-PL Models of Positive and Negative Items*

*
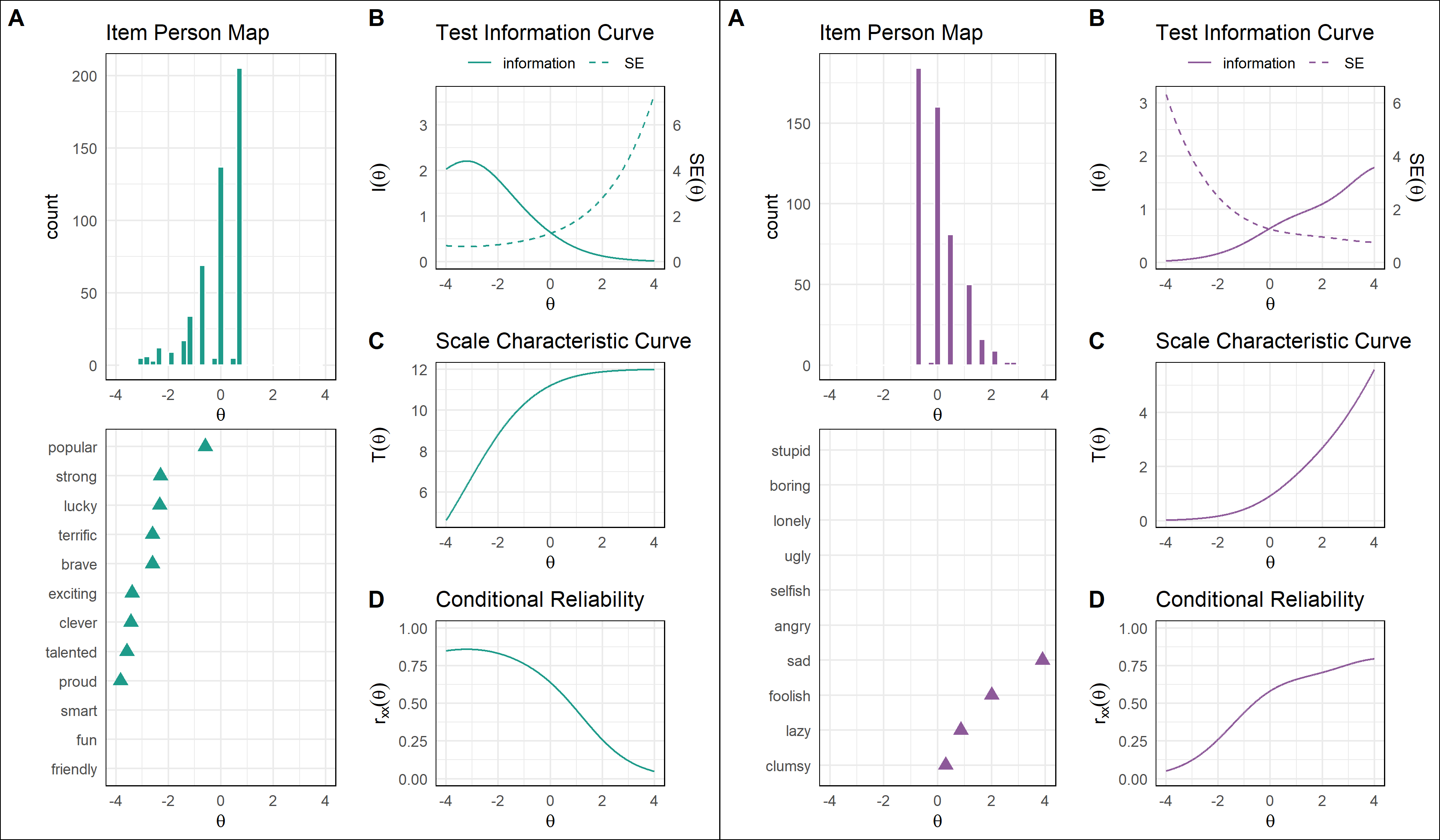
*

*Note.* SE = standard error. r_xx_ = conditional reliability. Theta (θ) = children’s underlying negative self-concept. (A-D) 1-PL model for positive item set. (E-H)1-PL model for negative item set. (A) Difficulty parameters were below -4 for the items *smart*, *fun*, and *friendly* in the 1-PL model for the positive item set and are therefore not shown. (E) Difficulty parameters were above 4 for the items *stupid, boring, lonely, ugly, selfish,* and *angry* in the1-PL model for the negative item set and are therefore not shown. The item *ashamed* was not included due to its poor fit and large *a* parameter, which distorted visualizations of summary statistics for the overall scale.

**Scale characteristic curves.** SCCs are plotted for the 1-PL model for the positive item set in Figure B2C. As in the 2-PL model, the number of items endorsed increased as theta values became less negative before plateauing at theta values over 1.0. This suggests that the number-endorsed score is a sufficient estimation of children’s positive self-concepts when positive self-concepts are in the below-average to average range. The rate of increase was somewhat smaller for the 1-PL model for the positive item set, indicating a weaker positive association between the total number of positive items endorsed and children’s overall positive self-concept compared to the 2-PL model.

SCCs are plotted for the 1-PL model for the negative item set in Figure B2G. The number of items endorsed increased as theta values became more positive before plateauing at theta values below -1.0, suggesting that the number-endorsed score estimates children’s negative self-concepts well when these are in the average to above-average range. The rate of increase was substantially smaller for the 1-PL model for the negative item set, indicating a weaker positive association between the total number of negative items endorsed and children’s overall negative self-concept compared to the 2-PL model.

**Conditional reliability.** Using a cut-off of .75, conditional reliability was best in the -4.0 ≤ θ ≤ -1.0 range for the 1-PL model for the positive item set (Figure B2D). In contrast, conditional reliability was best in the 3.0 ≤ θ ≤ 4.0 range for the 1-PL model for the negative item set (Figure B2H). Thus, reliability was poor at higher values of theta in the model of positive items and lower values of theta in the model of negative items.

***Difficulty and Discrimination Parameters***

ICCs and IICs for the 1-PL model for the positive item set are plotted in Figure B2. The order of items from most to least endorsed, as captured by their ICCs, generally mirrored the 2-PL model. That is, children were most likely to endorse *friendly* and least likely to endorse *popular*, though difficulty parameters were more negative in the 1-PL model overall.

**Figure B2**

*Item Characteristic and Information Curves for1-PL Model for Positive Item Set*

*
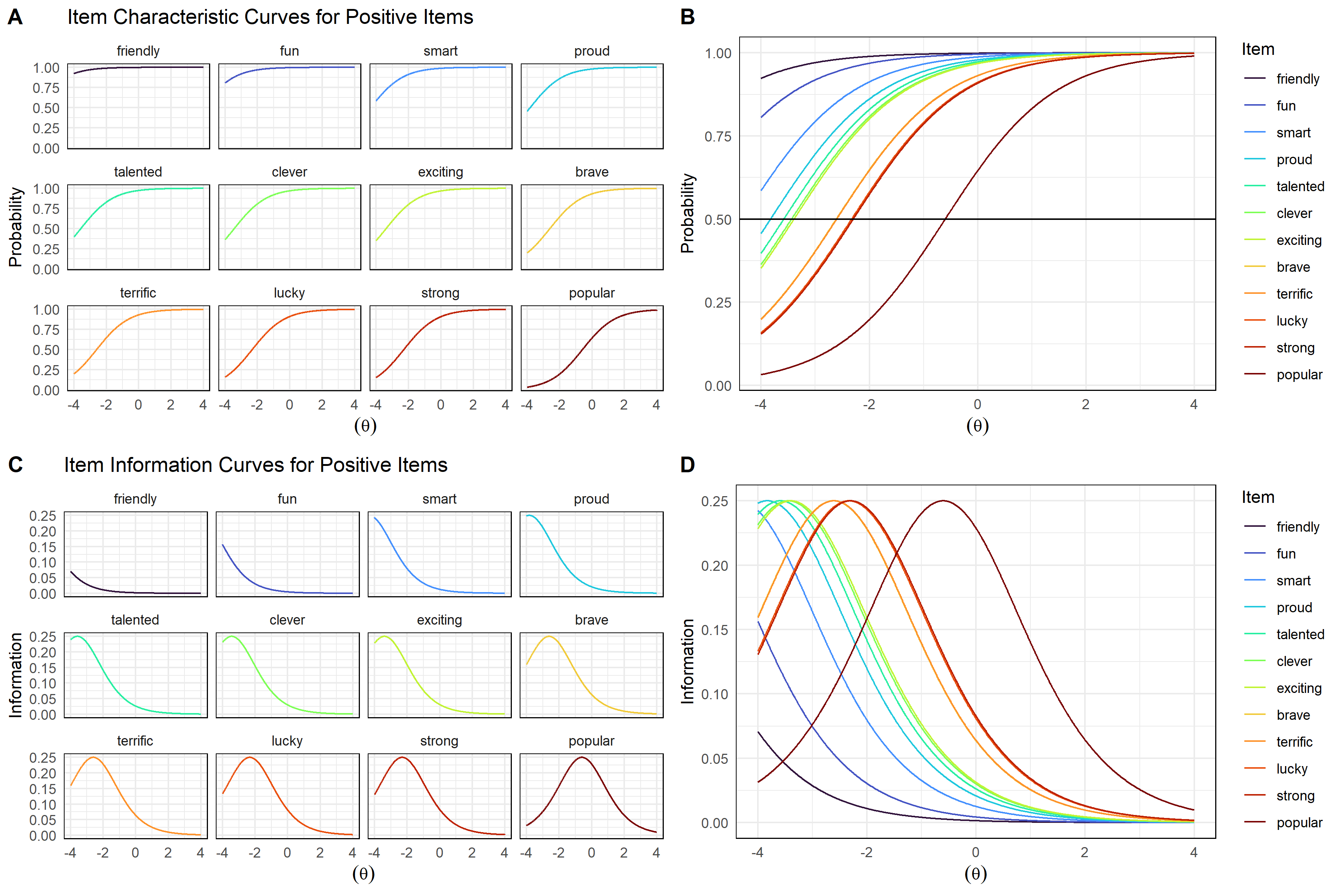
 Note.* Theta (θ) = Children’s latent positive self-concept. Item characteristic curves shown plotted individually (A) and together (B). Item information curves shown plotted individually (C) and together (D). In 1-PL models, where the discrimination parameter is held constant, ICCs do not intersect and all items have the same information and distribution.

ICCs and IICs for the 1-PL model for the negative item set are plotted in Figure B3. As with models of positive items, the rank order of items by difficulty was similar across the negative 1-PL and 2-PL models. Restricting variation in discrimination parameters resulted in substantially different ICCs compared to the 2-PL model for several items, including *angry*, *selfish*, *lonely*, *sad*, *stupid*, *boring*, and *ugly*. Difficulty parameters were substantially more positive for these items in the 1-PL model, such that their endorsement thresholds were much higher, occurring mostly at theta values greater than 4.0 as opposed to theta values between 2.0 and 3.0 greater in the 2-PL model. Thus, as in the 2-PL model, the 1-PL model indicates that most negative items appear best equipped for assessing children with highly negative self-concepts.

**Figure B3**

*Item Characteristic and Information Curves for 1-PL Model for Negative Item Set*


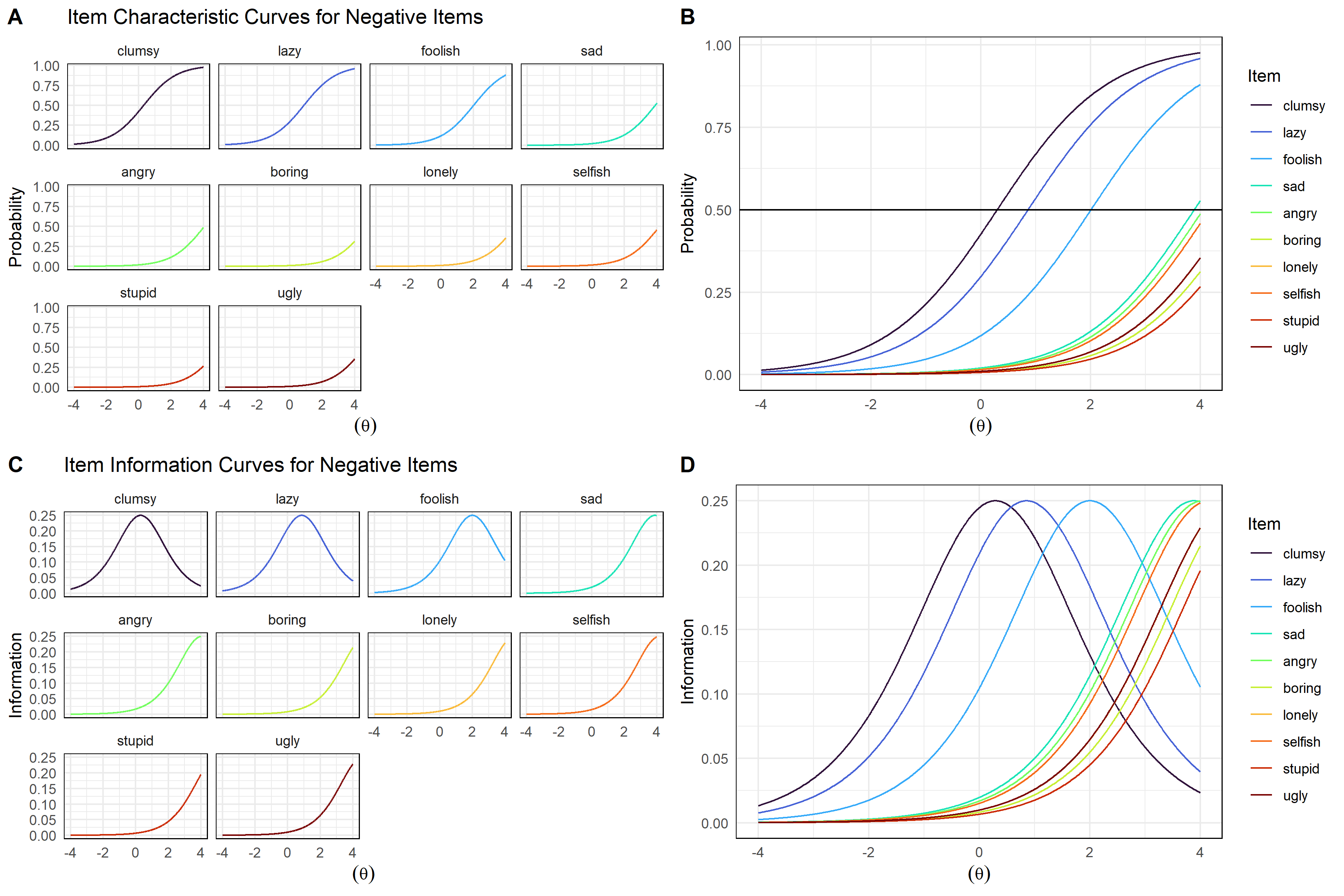


*Note.* Theta (θ) = Children’s latent negative self-concept. Item characteristic curves shown plotted individually (A) and together (B). Item information curves shown plotted individually (C) and together (D).

**Test information curve.** The TIC for the 1-PL model of the positive SRET scale is shown in Figure B2B. As in the 2-PL model, the positive SRET scale performs well when estimating negative scores, but has less precision when estimating theta scores greater than zero. Thus, the positive scale shows good estimation for positive self-concepts that fall below average, but performs poorly when these fall above average.

The TIC for the 1-PL models for the negative SRET scale is shown in Figure B2F. In contrast to the positive scale, the negative scale adequately estimates positive values of theta, but falters when estimating theta scores below zero. Thus, in contrast to the positive scale, the negative scale shows good estimation for high negative self-concepts and poor estimation for low negative self-concepts.

**Table S3**

*Parameter Estimates for1-PL Model of Positive Item Set*

|  |  | 1-PL | | | | |  |
| --- | --- | --- | --- | --- | --- | --- | --- |
| Item |  | *S*-χ^2^ (df) | *p* | RMSEA | *b* |  | |
| *Benchmark for good fit* |  | Non-significance | > .05 | .00 to .05 | – |  | |
| fun |  | 6.01* (1) | .014 | .10 | -5.42 |  | |
| terrific |  | 6.47 (5) | .263 | .02 | -2.61 |  | |
| exciting |  | 7.07 (6) | .315 | .02 | -3.39 |  | |
| proud |  | 8.10 (7) | .324 | .02 | -3.83 |  | |
| talented |  | 8.71 (6) | .190 | .03 | -3.58 |  | |
| smart |  | 5.95 (7) | .545 | .00 | -4.35 |  | |
| strong |  | 8.14 (5) | .129 | .04 | -2.30 |  | |
| brave |  | 8.49 (5) | .131 | .04 | -2.62 |  | |
| popular |  | 1.28 (3) | .724 | .00 | -.60 |  | |
| friendly^†^ |  | – | – | – | -6.49 |  | |
| clever |  | 4.96 (6) | .549 | .00 | -3.44 |  | |
| lucky |  | 18.57** (5) | .002 | .07 | -2.33 |  | |

*Note.* **p* < .05, ***p* < .01, ****p* < .001. SRET = Self-Referent Encoding Task. Parameter estimates are standardized.

^†^ Item fit statistics could not be calculated for *friendly*.

**Table S4**

*Parameter Estimates for 1-PL Model of Negative Item Set*

|  |  | 1-PL | | | |
| --- | --- | --- | --- | --- | --- |
| Item |  | *S*-χ^2^ (df) | *p* | RMSEA | *b* |
| *Benchmark for good fit* |  | Non-significance | > .05 | .00 to .05 | – |
| ashamed |  | 13.70*** (1) | .000 | .16 | 5.93 |
| ugly |  | 3.84 (4) | .428 | .00 | 4.64 |
| lonely |  | 5.60 (4) | .232 | .03 | 4.65 |
| boring |  | 3.97 (3) | .265 | .03 | 4.84 |
| angry |  | 4.90 (4) | .298 | .02 | 4.09 |
| stupid |  | .98 (3) | .805 | .00 | 5.06 |
| selfish |  | 6.22 (4) | .184 | .03 | 4.20 |
| lazy |  | 4.77 (2) | .092 | .05 | .86 |
| sad |  | .61 (4) | .962 | .00 | 3.92 |
| clumsy |  | 1.10 (1) | .295 | .01 | .30 |
| foolish |  | 9.21* (3) | .027 | .06 | 2.02 |

*Note.* **p* < .05, ***p* < .01, ****p* < .001. SRET = Self-Referent Encoding Task. Parameter estimates are standardized.

**Appendix D**

**Checking Assumptions**

**Unidimensionality**

Unidimensionality is the condition whereby all items of a measure reflect a single latent trait and is often determined via factor analysis and examining the fit of individual items. The unidimensionality of the SRET’s positive and negative factors is supported by the results of CFAs reported in the main manuscript, which showed good fit for a two-factor structure as well as good fit for separate models of the positive and negative factors.

**Local Independence**

Local independence is the condition whereby items are independent of each other and related only through the fact that they measure the latent trait. Thus, when accounting for the latent trait, there should be minimal correlation between items; in contrast, large and significant correlations between residuals suggest item nonindependence. Residual correlations should be considered relative to each other as well as the mean residual correlation, such that *Q_3_* residual correlations falling 0.2 units above the mean suggests local dependence (Marais, 2013). We inspected residual correlation matrices to identify potential violations of local independence. For the 1-PL model for the positive item set (mean *Q_3_* = -.06), residual correlations for three item pairs reached significance: *clever-smart* (*Q_3_* = .24)*, exciting-fun* (*Q_3_* = .19)*,* and *smart-talented* (*Q_3_* = .19). Of these, two pairs remained significant in the positive 2-PL model (mean *Q_3_* = -.06): *clever-smart* (*Q_3_* = .26) and *exciting-fun* (*Q_3_* = .15)*.* For the 1-PL model for the negative item set (mean *Q_3_* = -.04), residual correlations for two item pairs reached significance: *ashamed-lonely* (*Q_3_* = .25) and *lonely-ugly* (*Q_3_* = .17). Of these, one item pair remained significant in the negative 2-PL model (mean *Q_3_* = -.05): *lonely-ugly* (*Q_3_* = .17). In summary, there were few issues of local dependence across models. However, the positive items *smart, exciting*, and *fun* and the negative item *lonely* may be redundant with other items.

**Monotonicity**

Monotonicity is the phenomenon by which the probability of endorsing an item increases as an individual’s position on the latent trait increases. We examined the monotonicity of individual items using the *mokken* package in R (Van der Ark, 2007). Visual inspection of individual item step response functions and review of summary statistics showed no significant violations of monotonicity for any SRET item. Of note, monotonicity summary statistics could not be calculated for the positive item *fun* due to its highly skewed distribution (i.e., the vast majority of children endorsed this word as self-descriptive).

**References for Appendices**

Marais, I. (2013). Local dependence. In Christensen, K.B., Kreiner, S. & Mesbah, M. (Eds.), *Rasch models in health*. Hoboken, NJ: John Wiley & Sons Inc.

Stemler, S. E., & Naples, A. (2021). Rasch measurement v. item response theory: Knowing when to cross the line. *Practical Assessment, Research, and Evaluation*, *26*(11). https://doi.org/10.7275/v2gd-4441

Van der Ark, L. A. (2007). Mokken Scale Analysis in R. Journal of Statistical Software, *20*(11), 1–19. <https://www.jstatsoft.org/article/view/v020i11>.

1. We hereafter refer to the Rasch model as a 1-PL model for simplicity, while acknowledging the opposing aims of the Rasch and IRT traditions. [↑](#footnote-ref-1)
